# A genome-wide association for kidney function and endocrine-related traits in the NHLBI's Framingham Heart Study

**DOI:** 10.1186/1471-2350-8-S1-S10

**Published:** 2007-09-19

**Authors:** Shih-Jen Hwang, Qiong Yang, James B Meigs, Elizabeth N Pearce, Caroline S Fox

**Affiliations:** 1 National Heart Lung and Blood Institutes, Bethesda, MD, USA; 2 Massachusetts General Hospital and Harvard Medical School , Boston, MA, USA; 3 Department of Biostatistics, Boston University School of Public Health, Boston, MA, USA; 4 Boston University School of Medicine, Boston, MA, USA; 5 Department of Endocrinology, Diabetes, and Hypertension, the Brigham and Women's Hospital, Harvard Medical School, Boston, MA, USA

## Abstract

**Background:**

Glomerular filtration rate (GFR) and urinary albumin excretion (UAE) are markers of kidney function that are known to be heritable. Many endocrine conditions have strong familial components. We tested for association between the Affymetrix GeneChip Human Mapping 100K single nucleotide polymorphism (SNP) set and measures of kidney function and endocrine traits.

**Methods:**

Genotype information on the Affymetrix GeneChip Human Mapping 100K SNP set was available on 1345 participants. Serum creatinine and cystatin-C (cysC; n = 981) were measured at the seventh examination cycle (1998–2001); GFR (n = 1010) was estimated via the Modification of Diet in Renal Disease (MDRD) equation; UAE was measured on spot urine samples during the sixth examination cycle (1995–1998) and was indexed to urinary creatinine (n = 822). Thyroid stimulating hormone (TSH) was measured at the third and fourth examination cycles (1981–1984; 1984–1987) and mean value of the measurements were used (n = 810). Age-sex-adjusted and multivariable-adjusted residuals for these measurements were used in association with genotype data using generalized estimating equations (GEE) and family-based association tests (FBAT) models. We presented the results for association tests using additive allele model. We evaluated associations with 70,987 SNPs on autosomes with minor allele frequencies of at least 0.10, Hardy-Weinberg Equilibrium p-value ≥ 0.001, and call rates of at least 80%.

**Results:**

The top SNPs associated with these traits using the GEE method were rs2839235 with GFR (p-value 1.6*10^-05^), rs1158167 with cysC (p-value 8.5*10^-09^), rs1712790 with UAE (p-value 1.9*10^-06^), and rs6977660 with TSH (p-value 3.7*10^-06^), respectively. The top SNPs associated with these traits using the FBAT method were rs6434804 with GFR(p-value 2.4*10^-5^), rs563754 with cysC (p-value 4.7*10^-5^), rs1243400 with UAE (p-value 4.8*10^-6^), and rs4128956 with TSH (p-value 3.6*10^-5^), respectively. Detailed association test results can be found at http://www.ncbi.nlm.nih.gov/projects/gap/cgi-bin/study.cgi?id=phs000007. Four SNPs in or near the *CST*3 gene were highly associated with cysC levels (p-value 8.5*10^-09 ^to 0.007).

**Conclusion:**

Kidney function traits and TSH are associated with SNPs on the Affymetrix GeneChip Human Mapping 100K SNP set. These data will serve as a valuable resource for replication as more SNPs associated with kidney function and endocrine traits are identified.

## Background

Kidney disease affects 19 million adults in the United States [1]. Chronic kidney disease (CKD) is associated with cardiovascular disease [2-4], stroke [5], peripheral arterial disease [5,6], and all-cause mortality [7,8]. CVD risk factors are associated with the development of kidney disease [9], and the prevalence of traditional and novel CVD risk factors is elevated among those with kidney disease [7,10]. Urinary albumin excretion (UAE) is an early marker of kidney function that predicts CKD progression [11-14]. While glomerular filtration rate (GFR) and UAE are both measurements for kidney function, they represent different phenotypes and identify different subsets of at-risk individuals [15].

Genetic factors play a role in the progression of renal disease. Familial aggregation of end-stage renal disease has been identified [16]. Linkage analyses of kidney function have been conducted [17-21], and novel loci have been mapped to chromosomes 1 [18], 2 [21,22], 3 [17], 7 [22], 10 [19,20,22], and 18 [22]. In the Framingham Heart Study, we have shown that kidney function is heritable [23], suggesting a role for genetic mechanisms in its etiology. Results of the linkage study from the Framingham Heart Study suggested linkage between kidney disease and a locus on chromosome 4 with a LOD score of 2.2 [23]. Familial clustering of UAE has been observed in siblings of subjects with diabetes [24], and UAE has been shown to be heritable among the offspring of diabetic subjects [25]. Genome-wide linkage analyses have mapped novel loci to chromosomes 12 [26] and 19 [26] among families enriched for hypertension. Among families with more severe forms of nephropathy, suggestive evidence for linkage has been found on chromosome 10p [27] and 9q31–32 [28]. In the Framingham Heart Study, we observed a LOD score of 2.2 for UAE on chromosome 8 [29].

Thyroid disease, including Hashimoto's thyroiditis and Graves' disease, has a known familial component [30], and the same genes may underlie both conditions [31]. Measures of thyroid function have been shown to be heritable [32-34], and linkage has been reported to chromosome 18 for autoimmune thyroid disease in at least 2 studies [35,36].

As part of the Framingham Heart Study 100K Project, we sought to test the relation of multiple kidney and endocrine traits to 70,987 SNPs. In this manuscript, we focus the results of association studies for GFR, UAE, cysC, and thyroid stimulating hormone (TSH), a sensitive measure of thyroid function.

## Methods

Overall, 1345 participants were genotyped for the Affymetrix GeneChip Human Mapping 100K SNP set. For this manuscript, we focused on GFR from examination 7, UAE from examination 6, serum cysC from examination 7, and mean TSH from examinations 3 and 4. Phenotypes were available in 1010 participants for GFR at exam cycle 7, 822 participants for UAE at exam cycle 6, 981 participants for cysC at exam cycle 7, and 810 participants for mean TSH at exam cycles 3 and 4. Details about the selection process and genotyping are provided in the Overview [37]. Age-sex- and multivariable-adjusted residuals were generated; we present here only the results for multivariable-adjusted traits (all available results can be found in the website http://www.ncbi.nlm.nih.gov/projects/gap/cgi-bin/study.cgi?id=phs000007). We evaluated associations with 70,987 SNPs on autosomes with minor allele frequencies of at least 0.10, HWE p-value ≥ 0.001, and genotypic call rates of at least 80%.

### Phenotype assessment

Serum creatinine was measured using the modified Jaffe method at exams 2 (1978–1981), 5 (1991–1995), 6 (1995–1998), and 7 (1998–2001), and glomerular filtration rate (GFR) was estimated using the simplified Modification of Diet in Renal Disease Study equation [38,39]. CKD was defined based on the National Kidney Foundation Kidney Disease Outcome Quality Initiative working group, and modified slightly as previously described [9]. Urinary albumin concentration (UAE) was measured by immuno-turbidmetry (Tina-quant Albumin assay; Roche Diagnostics, Indianapolis, IN) during the sixth examination cycle (1995–1998). Urinary albumin was indexed to urinary creatinine (as the urine albumin/creatinine ratio, UACR) in order to account for differences in urine concentration. UACR is a validated and reliable single-sample measure of urinary albumin excretion and is highly correlated with albumin excretion rates assessed by 24-h urine collection [40,41]. Cystatin-C (cysC) was measured using particle enhanced immunonephelometry (Dade Behring BN 100 nephelometer; Dade Behring – Cystatin C reagent) with an inter-assay and intra-assay coefficient of variation of 3.3 and 2.4%, respectively. We have previously published correlates of CKD in the Framingham Heart Study, including hypertension, diabetes, smoking, obesity, and low HDL cholesterol [9,42].

TSH was measured using a chemoluminescence assay (London Diagnostics, Eden Prairie, Minn) with a lower limit of detection of 0.01 mU/L. Luteinizing hormone (LH), follicle stimulating hormone (FSH), and dehydroepiandrosterone sulfate (DHEAS) were measured as previously described [43,44]. Briefly, DHEAS concentrations were measured on serum samples via radioimmunoassay (Diagnostic Products Corp, CA). Calcium and phosphorous were measured at the second examination cycle using a standard colorimetric method (Roche Diagnostics, Alameda, CA), and uric acid was measured at the second examination cycle using an autoanalyzer with a phosphotungstic acid reagent.

### Genotyping

Genotyping was performed using the 100K Affymetrix GeneChip. Please see the Overview [37] for details.

### Statistical methods

Phenotypes used for the analysis were created by generating normalized residuals. We generated both age-sex adjusted and multivariable adjusted residuals for each trait. Table 1 shows the covariates included in the multivariable adjustment; all data in this manuscript represents the multivariable-adjusted traits. All association analyses were performed using the generalized estimating equations or family based association tests; details are provided in the Overview [37]. Methods to verify family structure, generate identity-by-descent for these 1345 participants with genotype information as well as the markers used for linkage analysis, is detailed in the Overview [37]. To assess the clustering of significance between each SNP and phenotypes that were repeatedly measured in several examination cycles (see the third table in this article), we generated the geometric mean of p-values for SNPs that fit the following criteria: at least 4 out of 6 p-values of <0.01 in GEE or FBAT analyses for 6 GFR traits (change in serum creatinine from exam 2 to 7; GFR at exam 2; GFR at exam 5; GFR at exam 6; GFR at exam 7; mean GFR exams 2, 5, 6, 7); one out of two UACR traits (UACR; UACR in a sample enriched for hypertension); three out of three of TSH traits (TSH at exam 3; TSH at exam 4; mean TSH at exams 3 and 4). Among the GFR traits, Pearson correlation coefficients ranged from 0.18 (p < 0.001) between GFR at exam 2 and exam 7, to 0.77 (p < 0.001) for the mean of GFR at exams 2, 5, 6, and 7 and GFR at exam 7. Linkage analysis was performed using the variance components methods on a subset of 100K markers and Marshfield short-tandem repeats; please see the Overview [37] for more details. Partial R^2^, the adjusted percentage of the phenotype variation explained by the genotype variation, was estimated by subtracting the adjusted R^2 ^value for a model that excludes the genotype from the R^2 ^value for a model that includes the genotype.

**Table 1:** Traits names, Framingham Heart Study examination cycle, and multivariable adjustments

Trait	Sample size	Exam cycle/s		
		Offspring	Original Cohort	Adjustment^†^

Serum Creatinine	840–1010	2, 5, 6, 7	0	Age and sex; multivariable*
Change in serum creatinine	854	2, 7	0	Age and sex; multivariable*
Glomerular Filtration Rate (GFR)	840–1010	2, 5, 6, 7	0	Age and sex; multivariable*
Chronic Kidney Disease	1010	7	0	Age and sex; multivariable*
Cystatin C	981	7	0	Age and sex; multivariable*
Uric acid	912–1031	1, 2	0	Age and sex; multivariable*
Urinary Albumin Excretion	822	6	0	Age and sex; multivariable*
Urinary Albumin Excretion ≥ 30 mg/g; Hypertension-enriched sample	532	6	0	Age and sex; multivariable*
Serum Calcium	906	2	0	Age and sex; age, sex, serum creatinine
Serum Phosphorus	911	2	0	Age and sex; age, sex, and serum creatinine
Thyroid stimulation hormone (TSH)	883	3	0	Age and sex; age, sex, body mass index, smoking, menopausal status, thyroid hormone use
Mean of TSH exam 3 & 4	810	3, 4	0	Age and sex; age, sex, body mass index, smoking, menopausal status, thyroid hormone use
Luteinizing hormone (LH) **	508	3	0	Age and multivariable***
Follicle stimulating hormone (FSH)**	509	3	0	Age and multivariable***
Dehydroepiandrosterone sulfate (DHEAS)	850	3	0	Age and sex; multivariable***

*Multivariable adjustment include age, sex, systolic blood pressure, hypertension treatment, HDL-cholesterol, smoking, diabetes, body mass index

**Men and post-menopausal women only with natural menopause not using hormone replacement treatment or oral contraceptive pills

*** Age, diabetes mellitus, impaired fasting glucose, smoking, systolic blood pressure, diastolic blood pressure, body-mass index, hypertension treatment, prevalent cardiovascular disease, total cholesterol/HDL ratio and alcohol intake

## Results

A description of all traits and phenotypes, including relevant examination cycles and multivariable-adjustments, is presented in Table 1. The median eGFR among individuals with CKD in our sample is 53.7 ml/min/1.73 m^2^. Table 2a presents the top 25 SNPs with the lowest p-values obtained via GEE for GFR, cysC, UAE, and mean TSH; additional results can be found on the National Center for Biotechnology Information website http://www.ncbi.nlm.nih.gov/projects/gap/cgi-bin/study.cgi?id=phs000007. The top SNP associated with GFR, cysC, and UAE were rs2829235 (p-value 1.6*10^-05^), rs1158167 (p-value 8.5*10^-09^) near the cysC precursor gene family (*CST*3, *CST*4, *CST*9), and rs1712790 (p-value 1.9*10^-06^), respectively (Table 2a). The top SNP to be associated with mean TSH was rs6977660 (p-value 3.7*10^-06^). Three SNPs were not shown on Table 2a due to the linkage disequilibrium (LD) (r^2 ^> 0.8) with the other top SNPs. These three SNPs were significantly associated with UAE at exam 6: rs9305355 (p-value 2.1*10^-5^) in LD with rs9305354, rs725304 (p-value 2.5*10^-5^) in LD with rs723464, and rs725307 (p-value 3.2 *10^-5^) in LD with rs723464. Table 2b presents the top SNPs based on the FBAT procedure. One SNP, rs10511594 (p = 9.0*10^-5^) was in LD (r^2 ^> 0.8) with rs7865184 (p = 4.0*10^-5^) for mean TSH.

**Table 2:** Most significant results for GFR (examination 7), UAE (examination 6), cysC (examination 7), and mean TSH (examinations 3 and 4) by GEE (2a), FBAT (2b) and linkage (2c) analyses

**2a. **Top 25 SNPs for association with GFR (examination 7), UAE (examination 6), cysC (examination 7), and mean TSH (examinations 3 and 4) based on the lowest p value of the GEE test. Corresponding phenotype names on the web are GFRMV7 (GFR), UAELNMV6 (UAE), CYSCMV7 (CysC), and TSHMEAN34MV (TSH)						
**TRAIT**	**SNP**	**Physical Location (Mb)**	**Chromosome**	**P value – FBAT**	**P value – GEE**	**GENE**

CysC	rs1158167	23,526,189	20	0.006	8.5*10^-09^	*CST9L|CST9|CST3*
UAE	rs1712790	114,126,679	11	0.014	1.9*10^-06^	*FAM55B*
TSH	rs6977660	19,578,720	7	0.010	3.7*10^-06^	
TSH	rs9322817	105,338,926	6	0.502	6.5*10^-06^	*HACE1*
TSH	rs10499559	21,882,699	7	0.068	8.3*10^-06^	*RAPGEF5*
UAE	rs9305354	28,397,067	21	0.013	8.4*10^-06^	
CysC	rs2145231	23,573,547	20	0.011	1.1*10^-05^	*CST9|CST3|CST4*
UAE	rs723464	133,940,196	4	0.000	1.1*10^-05^	
UAE	rs2113379	207,177,180	2	0.003	1.4*10^-05^	*ADAM23*
GFR	rs2839235	46,625,020	21	0.055	1.6*10^-05^	*PCNT2*
TSH	rs10493147	129,095,104	4	0.040	2.1*10^-05^	*HSPA4L*
TSH	rs784490	39,148,534	3	0.005	2.8*10^-05^	*TTC21A*
UAE	rs278021	6,698,929	1	0.958	2.9*10^-05^	*DNAJC11*
UAE	rs1856190	33,598,615	9	0.127	3.0*10^-05^	
UAE	rs10485409	91,562,132	6	0.147	3.1*10^-05^	
UAE	rs2785980	216,088,914	1	4.8*10^-04^	3.7*10^-05^	
UAE	rs837678	191,168,583	3	0.012	3.8*10^-05^	*LEPREL1*
GFR	rs3095160	49,844,269	13	0.002	3.8*10^-05^	
UAE	rs2761171	99,278,898	13	0.078	4.1*10^-05^	*CLYBL*
GFR	rs10507344	24,623,069	13	0.017	4.1*10^-05^	*PABPC3*
GFR	rs890945	157,924,801	5	0.036	4.7*10^-05^	
UAE	rs10502192	114,127,562	11	0.051	4.9*10^-05^	*FAM55B*
GFR	rs10489639	157,492,600	1	0.194	4.9*10^-05^	*CD48*
TSH	rs9308765	118,759,439	2	0.353	5.1*10^-05^	
TSH	rs3908399	12,849,275	20	8.0*10^-04^	5.2*10^-05^	

**2b. **Top 25 SNPs for association with GFR (examination 7), UAE (examination 6), cysC (examination 7), and mean TSH (examinations 3 and 4) based on the lowest p-value of the FBAT test. Corresponding phenotype names on the web are GFRMV7 (GFR), UAELNMV6 (UAE), CYSCMV7 (CysC), and TSHMEAN34MV (TSH)						

**TRAIT**	**SNP**	**Physical Location (Mb)**	**Chromosome**	**P value - FBAT**	**P value - GEE**	**GENE**

GEE	GENE					
UAE	rs1243400	9,016,664	10	4.8*10^-06^	0.036	
UAE	rs827640	9,028,017	10	1.5*10^-05^	0.047	
UAE	rs7315682	32,577,224	12	2.1*10^-05^	0.28	*FGD4*
GFR	rs6434804	196,589,899	2	2.4*10^-05^	0.003	*DNAH7*
UAE	rs1543468	230,655,160	1	2.5*10^-05^	0.253	*SLC35F3*
UAE	rs723464	133,940,196	4	2.7*10^-05^	1.0*10^-05^	
TSH	rs4128956	131,848,063	9	3.6*10^-05^	0.355	
TSH	rs7865184	14,677,867	9	3.9*10^-05^	0.082	*ZDHHC21*
CysC	rs563754	61,068,849	18	4.7*10^-05^	0.002	
GFR	rs2885618	41,244,839	18	4.8*10^-05^	1.0*10^-04^	
TSH	rs701801	100,166,859	10	5.8*10^-05^	0.090	*HPS1*
UAE	rs33855	171,124,642	5	6.2*10^-05^	0.192	
GFR	rs10483741	61,059,454	14	6.3*10^-05^	0.298	*PRKCH*
CysC	rs2121267	46,461,040	2	6.6*10^-05^	0.002	*EPAS1*
CysC	rs1400306	83,256,163	11	7.2*10^-05^	0.061	
TSH	rs1223531	13,255,521	6	8.0*10^-05^	0.123	*PHACTR1*
CysC	rs10504244	59,147,328	8	8.3*10^-05^	0.005	
UAE	rs2374688	106,175,669	12	9.1*10^-05^	0.087	*BTBD11*
UAE	rs10519012	58,333,458	15	1.0*10^-04^	0.088	
CysC	rs1931619	82,682,388	6	1.0*10^-04^	0.05	
UAE	rs10501264	41,387,406	11	1.1*10^-04^	7.2*10^-04^	
UAE	rs10492025	111,735,770	12	1.1*10^-04^	0.41	*RPH3A*
UAE	rs2056694	128,517,768	7	1.1*10^-04^	0.1	
UAE	rs8113386	16,332,871	19	1.1*10^-04^	0.053	*KLF2*
TSH	rs295136	200,966,508	2	1.3 *10^-04^	0.64	

**2c. **Magnitude and Location of Peak LOD scores for regions in which LOD exceeds 2.5.* Corresponding phenotype names on the web are PHOSPHORUSMV2 (serum phosphorous), URICACIDMV2 (uric acid), SCRLNMVNL6 (serum creatinine exam 6), LHMV3 (luteinizing hormone), GFRMVNL6 (GFR exam 6), CALCIUMMV2 (serum calcium), GFRMV7 (GFR exam 7), and SCRLNMVNL5 (serum creatinine exam 5)						

**Trait**	**SNP**	**Chromosome**	**Physical location (Mb)**	**LOD 1.5 (Lower; Mb)**	**LOD 1.5 (Upper; Mb)**	**LOD**

Serum Phosphorous	rs754958	8	134307953	130413367	139409406	4.33
Uric acid	rs10495487	2	2310945	107241	3787803	4.28
Serum creatinine (exam 6)	rs10489578	1	229251990	224108350	230301885	3.35
Luteinizing Hormone	rs10515134	17	52218447	45043525	59136652	3.17
GFR (exam 6)	rs10489578	1	229251990	220881707	230301885	3.08
Serum Calcium	rs10484370	6	18686366	10181754	21792915	3.03
GFR (exam 7)	rs10511176	3	100809191	73551217	103238533	2.79
Serum creatinine (exam 5)	rs10502302	18	2542886	156277	3857290	2.51

*All traits are multivariable-adjusted; see Table 1 for specific covariate adjustments.

Table 2c presents all traits examined with LOD scores of at least 2.5. One locus on chromosome 1 (nearest marker on the 100K GeneChip, rs10489578) was linked to GFR with a LOD score of 3.08. We observed a LOD score of 4.28 for uric acid to chromosome 2 (nearest marker rs10495487), a location we have previously identified using Marshfield linkage analysis to uric acid [45].

Table 3 presents the top SNPs for our multiple phenotype analysis for GFR, UAE, and TSH with a total of 24 SNPs showing consistently significant associations with multiple related phenotypes. Tables 4a and 4b present results looking at replication of genes that have been associated with kidney traits in the published literature. Four SNPs in or near the *CST*3 gene were highly correlated with cysC levels (p-value 8.5*10^-09 ^to 0.007). All four SNPs have minor allele frequencies greater than 10% and none were in linkage disequilibrium (defined by R^2 ^> 0.8) as shown on Table 4. The proportion of the cysC variation that can be explained by these SNPs is shown in Table 4. rs1158167 accounts for 2.5% of the cysC variation. We found nominal significance between a SNP near the *APOE *gene and CKD (p = 0.04).

**Table 3:** SNPs showing the top 8 significant association with multiple measurements of GFR, UACR, or TSH phenotypes.* Corresponding phenotype names on the web are GFRMV7 (GFR), UAELNMV6 (UAE), and TSHMEAN34MV (TSH).

Trait	chromosome	SNP (rsID)	Physical Location	Genes (in or near)	Mean p-value (GEE)	Mean p-value (FBAT)
GFR	21	rs2839235	46625020	*PCNT2*	6.3*10^-4^	0.281
GFR	17	rs10512437	27046466		0.002	0.197
GFR	13	rs2480555	70785310	*DACH1*	0.003	0.006
GFR	7	rs10486135	11301740		0.004	0.142
GFR	7	rs727087	8244570	*ICA1*	0.004	0.223
GFR	13	rs1005066	70790573	*DACH1*	0.004	0.022
GFR	18	rs2885618	41244839	*SETBP1*	0.004	0.024
GFR	2	rs10496887	142198571	*LRP1B*	0.005	0.091
UAE	11	rs1712790	114126679	*FAM55D*	9.1*10^-07^	0.009
UAE	6	rs10485409	91562132	*EPHA7*	1.0*10^-05^	0.067
UAE	21	rs9305354	28397067		1.9*10^-05^	0.018
UAE	11	rs10502192	114127562	*FAM55D*	3.6*10^-05^	0.041
UAE	1	rs2077678	75246848		4.4*10^-05^	0.022
UAE	4	rs723464	133940196		4.9*10^-05^	0.000
UAE	21	rs9305355	28397088		5.0*10^-05^	0.011
UAE	6	rs10484587	143183270	*AIG1*	5.2*10^-05^	0.032
TSH	7	rs6977660	19578720		1.6*10^-05^	0.022
TSH	4	rs10493147	129095104	*HSPA4L*	2.1*10^-05^	0.019
TSH	7	rs10499559	21882699	*DNAH11*	2.8*10^-05^	0.111
TSH	6	rs9322817	105338926		7.4*10^-05^	0.576
TSH	2	rs9308765	118759439	*INSIG2*	7.7*10^-05^	0.404
TSH	6	rs6942231	105298507		1.6*10^-04^	0.541
TSH	7	rs10486365	19574604		1.9*10^-04^	0.221
TSH	7	rs10486653	34484903	*BMPER*	2.7*10^-04^	0.252

*see details in methods for criteria for generating mean p-value

**Table 4:** Results on Association Analysis for Candidate Genes

**4a. **Results of GEE analysis between SNPs in the *CST*3 and *APOE *candidate genes and the kidney function traits with p-value < 0.05. Corresponding phenotype names on the web are CYSMV7 (CysC) and CKDMV7 (CKD).							
**Candidate gene**	**PHENOTYPE**	**SNP**	**CHROMOSOME**	**Location**	**Minor allele frequency(%)**	**P_value (GEE)**	**Partial R^2^**

*CST3*	CysC	rs1158167	20	23,526,189	21	8.0 *10^-9^	2.5
*CST3*	CysC	rs2145231	20	23,573,547	15	1.1*10^-5^	1.2
*CST3*	CysC	rs726217	20	23,532,116	38	3.1*10^-4^	0.8
*CST3*	CysC	rs911122	20	23,573,746	37	0.007	1.1
*APOE*	CKD	rs3760626	19	50,148,945	45	0.04	-

**4b. **Results of FBAT analysis between SNPs in the *CST*3 candidate gene and kidney function traits with p-value < 0.05. Corresponding phenotype names on the web are CYSMV7 (CysC) and UAEGE30HTNMV6 (UAE ≥ 30 mg/g)							

**Candidate gene**	**PHENOTYPE**	**SNP**	**CHROMOSOME**	**Location**	**P-value**		

*CST3*	CysC	rs1158167	20	23,526,189	0.006		
*CST3*	CysC	rs2145231	20	23,573,547	0.011		
*CST3*	UAE ≥ 30*	rs911122	20	23,573,746	0.032		

*In a sample enriched for hypertensive individuals

### Additional findings

We also identified several other plausible candidate genes that appear in our list of top 500 SNPs for each trait (see http://www.ncbi.nlm.nih.gov/projects/gap/cgi-bin/study.cgi?id=phs000007). For GFR, we identified *LRP1B *(GEE p-value = 0.0006, rs1049688), *ADRBK2 *(GEE p-value = 0.002, rs1048312), *APOB *(GEE p-value = 0.003, rs1048312), several genes in the chromosome 17 cytokine gene cluster including *CCL3*, *CCL4*, and *CCL18 *(GEE p-value 0.004, rs1818816), *SCARB1 *(GEE p-value = 0.004, rs1902569), *NFKB1 *(FBAT p-value = 0.001, rs230489), *TGFB1 *(FBAT p-value = 0.003, rs2072239), and *PPARG *(FBAT p-value = 0.005, rs709157). For UAE, we also identified a SNP in the *LRP1B *gene (GEE p-value = 0.004, rs1049687). For cysC, we identified SNPs in the *LRP1B *gene (GEE p-value = 0.001, rs1463615), *ANGPT1 *(GEE p-value = 0.005, rs4354281), *NFKB1 *(FBAT p-value = 0.004, rs2991716), and *PPARG *(FBAT p-value = 0.004, rs1051041). For mean TSH, we identified SNPs in *ADRBK2 *(GEE p-value = 0.002, rs3888397), *TRHDE *(GEE p-value = 0.002, rs2044305) and *DIO2 *(GEE p-value = 0.003, rs54566), the *SCD*4 gene (rs10516679 GEE p-value = 0.0007 FBAT p-value = 0.02), *VLDLR *(FBAT p-value = 0.002, rs4084415) and *APOBEC2P *(FBAT p-value = 0.005, rs722442).

## Discussion

In our analysis of kidney-related traits, we have found strong evidence for association between multiple kidney-related traits and TSH with SNPs on the Affymetrix 100K GeneChip. We found strong evidence for association between cysC levels and 4 SNPs in or near the *CST*3 gene. For UAE, we observed strong association with *ADAM23*, a gene involved in the metalloproteinase family, which may be involved in the pathophysiology of glomerulosclerosis [46], and *PCDH9*, a gene that is a member of the cadherin superfamily. For TSH, we observed significant association with the *HSPA4L *gene with a mean p-value for all three TSH measurements, a gene that is part of the heat shock protein family, which may be involved in the pathophysiology of thyroid disease [47]. We also observed association with the *SCD*4 gene, a gene involved in the conversion of saturated to monounsaturated fatty acids; TSH is an important correlate of lipid levels [48].

In our linkage results, we observed a region we have previously noted for uric acid [45], albeit with a significantly higher LOD score. We identified a LOD score of 2.78 on chromosome 3, approximately 18 Mb away from a region previously noted in association with kidney function in hypertensive individuals [17], a region that lies within our 1.5 support LOD interval. We also report novel loci for GFR and TSH.

We show significant association between cysC levels and the *CST*3 gene, an observation that has been previously noted [49]. Our top SNP reaches genome-wide significance, and may represent a true finding. In our candidate gene approach, we found nominal significance for a SNP near the *APOE *gene, a gene that has been associated with CKD [50]. Unfortunately, poor coverage of the *APOE *gene by the Affymetrix 100K Genechip precluded a more in-depth test of association with SNPs in the *APOE *gene and CKD.

Strengths of our study lie in our assessment of multiple measures of kidney function and endocrine traits in a sample unselected for these traits, thus reducing bias. We also have excellent assessment of potential confounders that we are able to adjust for in our residual creation. Because the Framingham Heart Study has measured multiple traits, we are able to examine phenotype clustering. Limitations exist as well. Kidney function was ascertained by a single serum creatinine measure, which may lead to misclassification. Our sample was not selected for CKD, and as a result, affected individuals had moderate CKD as reflected by the median eGFR of 53.7 ml/min/1.73 m^2 ^among participants with CKD. The MDRD equation, which was used to estimate GFR, has been shown to underestimate GFR by 29% in healthy individuals [51]; therefore, we may have introduced additional misclassification into our trait definition. We used a spot urine specimen to assess UAE instead of a 24-hour collection. However, spot UAE approximates 24-hour collections [40], and are not prone to the error inherent in collecting 24-hour urine specimens. We used cysC as a continuous trait and did not use transforming equations to estimate GFR, as most existing equations have been developed in small, selected samples [52,53], or developed using immunoturbimetric method [53,54] instead of nephelometry and therefore we did not feel as though they were appropriate for use in our large population-based cohort. Further, we used cystatin C as a marker of kidney function but can not rule out that it may also reflect cardiovascular disease risk above and beyond its relation to kidney function [55-59]. Our focus on multivariable models may have led us to miss important bivariate associations between SNPs and measures of kidney function. Given that our findings have not yet been replicated, many p-values may represent false positive findings. We used TSH as an indicator of thyroid function, as we do not have measures of free thyroxine or a reliable assessment of thyroid disease in our study sample. Our sample is neither ethnically diverse nor nationally representative, and it is uncertain how our results would apply to other ethnic groups. However, in genetics studies, sample homogeneity is beneficial in order to reduce population stratification. For limitations pertaining to our genotyping or statistical methods, please see the Overview [37].

## Conclusion

Kidney function traits and TSH are associated with SNPs on the Affymetrix 100K SNP GeneChip. Replication of association between these traits and SNPs requires follow-up in independent samples. These data will serve as a valuable resource for replication as more SNPs associated with kidney function and endocrine traits are identified.

## Abbreviations

CKD = chronic kidney disease; cysC = cystatin-C; DHEAS = dehydroepiandrosterone sulfate; FBAT = family-based association tests; FSH = follicle stimulating hormone; GEE = generalized estimating equations; GFR = glomerular filtration rate; LD = linkage disequilibrium; LH = luteinizing hormone; MDRD = Modification of Diet in Renal Disease; SNP = single nucleotide polymorphism; TSH = thyroid stimulating hormone; UAE = urinary albumin excretion.

## Competing interests

The authors declare that they have no competing interests.

## Authors' contributions

SH generated the phenotype data, participated in the analysis, and drafted the manuscript. CF helped generate the phenotype data, interpret the results, and draft the manuscript. QY generated the phenotype data, interpreted the results, and helped draft the manuscript. JBM helped generate the phenotype data, interpret the results, and revised the manuscript critically for important intellectual content; and has given final approval of the version to be published. EP assisted in the acquisition and cleaning of the TSH data and critically reviewed a draft of the manuscript. All authors gave final approval to the manuscript.
